# Supplementary material for: Clinical Predictors and Prognosis of Myocardial Infarction with Non-Obstructive Coronary Arteries (MINOCA) without ST-Segment Elevation in Older Adults
Source: J Clin Med. 2023 Feb 2;12(3):1181. doi: 10.3390/jcm12031181 (PMC9918164; doi:10.3390/jcm12031181)
Supplement: Supplementary file 1 [file jcm-12-01181-s001.zip › jcm-2137204-supplementary.pdf]

Supplementary Material

Table S1. Clinical variables associated to all-cause mortality. Univariate analysis.

	HR (IC 95%)	p-value
**Age (years)**	1.07 (1.04-1.10)	<0.001
**Female sex (%)**	1.2 (0.87-1.7)	0.28
**High BP (%)**	1.2 (0.75-1.9)	0.44
**Dyslipidemia (%)**	0.96 (0.68-1.3)	0.79
**Diabetes (%)**	1.5 (1.10-2.1)	0.01
**Smoker (%)**	1 (0.58-1.9)	0.89
**Family history CAD (%)**	1.8 (0.45-7.4)	0.39
**Prior MI (%)**	1.2 (0.81-1.8)	0.34
**Prior PCI (%)**	1.5 (1-2.4)	0.04
**Peripheral artery disease (%)**	2.4 (1.4-4)	0.001
**Prior stroke (%)**	1.9 (1.1-3)	0.01
**Prior HF (%)**	1.5 (0.78-3)	0.21
**AF (%)**	2.5 (1.6-3.8)	0.001
**ST descent (%)**	1.8 (1.2-2.5)	0.001
**Negative T wave (%)**	1.8 (1.2-2.5)	0.17

**LBBB (%)**	1.6 (0.98-2.7)	0.05
**PM Rhythm (%)**	0.53 (0.17-1.7)	0.27
**Systolic BP (mmHg)**	0.99 (0.99-1)	0.03
**Diastolic BP (mmHg)**	0.98 (0.97-0.99)	0.002
**Heart rate (bpm)**	1.009 [0.99-1.02]	0.2
**Killip (≥2)**	2.1 (1.4-3.2)	0.003
**Hemoglobin (g/dL)**	0.79 (0.73-0.84)	<0.001
**White blood cells ($x10^6$/L)**	1.00 (1.00-1.00)	0.2
**Creatinine (mg/dL)**	2.20 (1.34-3.61)	0.002
**GFR (mL/min/m)**	0.97 (0.96-0.98)	<0.001
**Peak troponin level (ng/L)**	1.00 [1.00-1.00]	0.7
**LVEF**	0.97 [0.965-0.99]	<0.001
**LVEF<55% (%)**	0.67 [0.49-0.92]	0.013
**Wall motion abnormalities**	1.38 [1.02-1.88]	0.038
**ASA (%)**	0.87 [053-1.44]	0.599
**Clopidogrel (%)**	1.55 [1.07-2.25]	0.019
**DAPT (%)**	1.42 [0.99-2.01]	0.055
**ACE inhibitors (%)**	0.67 [0.48-0.93]	0.01

**BBK (%)**	1.3 [0.94-1.9]	0.12
**Statin (%)**	1.94 [0.68-1.3]	0.72

Abbreviations. ACE= angiotensin converting enzyme; AF= atrial fibrillation; ASA= acetylsalicylic acid; BBK= betablockers; BP= blood pressure; CAD= coronary artery disease; CV= cardiovascular; DAPT= dual antiplatelet therapy; GFR= glomerular filtration rate; HF= heart failure; LBBB= left bundle branch block; LVEF= left ventricle ejection fraction; MACE= major cardiovascular events; MI= myocardial infarction; PCI= percutaneous coronary intervention; PM= pacemaker.
